# Editorial

**Published:** 1862-08

**Authors:** 


### Editorial.

CHICAGO MEDICAL SOCIETY.—OVARIOTOMY.—DISEASES AT CAMP DOUGLAS.—CAMP DYSENTERY, &C.—The Chicago Medical Society held its regular monthly meeting in Larmon's block, September 5, 1862. The President, Dr. S. WICKERSHAM, was in the Chair, and a goodly number of the members were in attendance. Among the items of interest presented to the meeting we note the following:—

OVARIOTOMY.—Dr. A. FISHER presented an ovarian tumor, consisting of a single cyst of considerable size, and a solid part weighing about three pounds. The cyst had contained about sixty-four ounces of fluid. No part of the tumor exhibited any of the appearances of a cancerous or malignant structure, but the solid part evidently belonged to the class of simple fibrous growths. The patient from whom it had been removed was a married woman, 47 years of age, good constitution, and had borne four children. A small tumor was first felt in the right ileac region about one year since. In a short time it seemed to disappear from that side, and another made its appearance in the corresponding region of the left side, where it remained, and increased steadily until removed by the operation.

At the time the patient came under Dr. FISHER'S care for an operation, the abdomen was much distended with fluid, a great part of which was evidently in the cavity of the peritoneum. The tumor, however, was well defined, and very movable in all directions, showing the probable entire absence of adhesions. This, with the rapidity of its growth, and good constitution and health of the patient, caused the operation to be recommended without hesitation. The patient assenting, the operation was performed by Dr. FISHER, in the presence of Drs. MYERS, ALLEN, WICKERSHAM, and others.

On opening the peritoneum, about two gallons of water escaped from the cavity. The incision was then enlarged, the sac of the tumor punctured and relieved of two quarts of a serous fluid. The whole tumor was then found to be free from adhesions, and was readily drawn through the opening. A ligature was placed firmly around its root or pedicle, and the tumor excised.

The operation was finished by closing the wound with sutures which passed through the integument and the pedicle, so as to retain the latter at the surface.

Two grains of opium were administered and the patient placed in bed. No bad symptoms followed, and the patient recovered perfectly in a few weeks.

DISEASE IN CAMP DOUGLAS.—Dr. M. O. HEYDOCK, one of the assistant-physicians to the camp-hospitals, in which are a large number of rebel prisoners, made an interesting report in relation to the diseases that had been most prevalent among them during the past summer. Pneumonia, which had been so prevalent and fatal during the early Spring, had not entirely disappeared during the Summer, but was not of frequent occurrence.

The diseases most prevalent, among the prisoners, during the past three months, have been diarrhœa, dysentery, erysipelas, fevers, and dropsy. Erysipelas had been very prevalent, attacking almost every part of the cutaneous surface, in different cases, and presenting every degree of severity. It had been most successfully treated, by nutritious diet, and the internal use of from 20 to 40 drop doses of muriated tincture of iron, sometimes aided by quinine and opiates.

Most of the cases of fever, were of the continued or typhoid type. A few had seemed to possess an extreme degree of malignancy, running to a fatal termination in from 20 to 72 hours.

These rapidly fatal cases did not present the symptoms that usually characterize pernicious fever of a malarious origin. The patients had usually felt slightly indisposed for two or three days previous to the attack; when they would suddenly become overwhelmed with exhaustion, and on being brought into the hospital were found with face suffused with a dark flush, eyes injected, lips leaden color, the capillaries of the cutaneous surface generally congested, and the extremities cold. The pulse small, soft, and weak; respiration sometimes hurried, and at others slow and sighing; general restlessness; and dulness of mental faculties. In many cases the tongue was perfectly clean, and no evacuations, either by mouth or rectum, and little or no secretion of urine. In some cases the patients complained of very severe pain or distress, generally in the epegastric and hypochondriac regions, though sometimes in the head and back. The circulation became rapidly more feeble, the respiration less efficient, the coldness more general, a cold sweat appeared on the surface, and death occurred, in some cases, within twelve hours, and in others, not until the end of the third day. The appearance, on making *post mortem* examinations, were, general venous congestions or fulness of the venous capillaries, and the dark color and uncoagulated condition of the blood. The latter were constant appearances; the blood in large vessels and cavities of the heart being always dark, venous color, and entirely free of any coagula. The muscular structure of the heart was darker color and softer than natural. The liver was moderately enlarged in most cases, and its central portions decidedly softened, with points of black blood starting out from the surface whenever it was cut through. The spleen was generally found altered in the same direction, though not to the same extent as the liver. The lower and posterior portions of the lungs were usually moderately engorged with dark blood. No marked lesions in the intestines. The bladder was usually found empty, and the kidneys normal in size and only moderately congested with blood. Their structure appeared slightly softened, and the investing membrane more easily peeled of than natural. Neither the brain or spinal cord were examined in any of the cases. Neither did Dr. HEYDOCK make any mention of the appearances of the ganglia of the sympathetic nerve.

From the symptoms during life, and the appearances of the blood, as well as the general tendency to softening of structures, revealed by the *post mortem* examinations, we should regard these cases as belonging to the class of malignant typhus.

Both the predisposing and exciting causes were such as would be likely to engender this variety of fever.

Confinement for several months, as prisoners closely within the camp; a limited *variety* of food; and more or less mental depression, were well calculated to depress the vital properties, impoverish the blood, and enfeeble all the organic movements.

The system brought to this condition by such causes, and then exposed to the hot, sultry, and damp atmosphere of many of our summer days, was well calculated to so far suspend the vital affinity of one organic atom for another as to arrest organic change, and consequently both capillary circulation, secretion, and the evolution of caloric. Of course, such a condition leaves scarcely an available degree of susceptibility to the action remedies of any kind, or a capacity for reaction, and consequently the patients almost uniformly die in from half-a-day to three days from the commencement of the attack.

Dr. HEYDOCK told us that many remedies had been tried, chiefly belonging to the class of diffusable stimulants, though quinine had also been faithfully tried, in every variety of dose, from the smallest to the largest, but without any apparent effect whatever. He thought large doses of muriated tincture of iron, given as in the treatment of erysipelas, and aided by diffusable stimulants, had more effect than any other remedies tried.—Query: Would not the sudden application of cold effusions, alternated with warm dry flannel to the surface, and the liberal internal use of the chlorate of potassa or soda, in addition to the tincture of the chloride of iron, be more effectual in reviving the almost suspended susceptibility and vital affinity in such cases, and thereby establish a reliable reaction, than the means generally resorted to?

*Diarrhœa and Dysentery.*—Dr. Heydock stated that the cases of diarrhœa and dysentery were numerous; generally persistent, with a tendency to continue in a chronic form, accompanied by an extremely anemic condition of the blood, and frequently dropsical effusions. The symptoms of the more recent cases of dysentery differed from those usually presented, chiefly in there being less heat of skin, more palor, and a remarkable clean tongue, but little disposition to become either dry or red. This clean and rather pale appearance of the tongue and gums continued throughout the whole course of the disease, even in its most chronic form.

The *post mortem* appearances, observed in those who had died after a protracted course of the disease, were highly interesting. The adipose and muscular structures were in a state of extreme atrophy; even the heart itself was found much smaller than natural and of a paler color.

Serous effusions were found in most cases, and in some they existed in almost every cavity or serous sac in the body. But while the cavity of the peritoneum, pleura, pericardum, etc., were often found more or less distended with serous fluid, the presence of plastic exudations, adhesions, or any other traces of inflammation were very rarely observed. Purple or petechial spots were often found on that part of the peritoneum covering the colon and lower part of the ileum, and the usual appearances of readness, tumefaction, softening and ulceration in the mucous lining of the same portions of the alimentary canal, were uniformly present, to a greater or less extent.

Among a large variety of remedies used in the treatment of chronic cases, Dr. Heydock found the combination of alum and opium more efficient than any other in arresting the discharges. We have occasionally used alum in cases of dysentery, occurring in anemic subjects, for several years past, and generally with prompt benefit. In some cases, of protracted duration, in soldiers and officers returned from the army, during the present summer, a powder of alum, 5 grs., and opii. from 1 to 2 grs., given every six hours, with 15 drops of the liquor ferri nitratis between, has speedily overcome the disease.

In other cases, we have used the emulsion of oil of turpentine and laudanum every two, three, or four hours, according to the frequency of the discharges, and a pretty full dose of tannate of quinine every morning, with perfectly satisfactory results.

In some of this class of cases, in which there seems to be great relaxation of tissues, as well as impoverishment of the blood, the following formula has been productive of much good:

℞.—Strychnia,........................................ 1 gr.

Nitric Acid, ....................................... ʒj.

Tinct. Opii.,....................................... ʒjj.

Water, .............................................. ℥jj.

Mix, and give to an adult, a fluid drachm every 4 or 6 hours, diluted with sweetened water.

---

Prospects of our Medical Schools.—Our readers will be glad to know that the prospect of having a full class of students in attendance at the opening of the annual winter term, in the Medical Department of Lind University, is good; and all the arrangements are completed for having the instruction in each department full and complete. The dissecting room will be open, and material supplied for dissection, on or before the 1st of October; and the same liberal clinical advantages afforded as in former years.

There will be four hospital and two dispensary clinics each week, thus affording to the advanced students one hour of direct clinical instruction at the bedside of the sick every day.

The regular winter term commences on the second Monday in October, and continues until the first Monday in March.

The Rush Medical College commences its regular course on the 1st of October, and continues sixteen weeks.

Of the detailed arrangements for the term, and the prospects in reference to the number expected to attend, we are not informed, but presume both are substantially the same as in former years.

---

TREATMENT OF EPILEPSY.—Dr. John W. Ogle, assistant-physician to St. George Hospital, London, has an article in the September number of the *London Lancet*, calling attention to a species of the *galium* as a remedy for the cure of Epilepsy.

It seems that there has been, for several years past, an establishment at Tain, in the South of France, at which large numbers of Epileptic patients are treated annually, and with much reputed success. Almost the only medicine used is the expressed juice of a species of the galium. Dr. Ogle calls it the galium alba.

From the observations made by Dr. Ogle, and the testimony of others, it would appear that the remedy is worthy of further trial.

---

### OBSERVATIONS ON THE EFFECT OF THE PREPARATIONS OF IRON.

TRANSLATED FROM THE GERMAN

BY D. S. GANS, M.D., Cincinnati, Ohio.

---

Dr. Pakrowsky, of St. Petersburgh, had opportunity to observe several patients in the hospital there, who, for various diseases, took iron; and he directed his particular attention to the effect of that remedy on the tissue-change. To that end he measured daily in all the patients the temperature of the body, the quantity of the consumed food, the quantity of the excrements; of the urine, the specific gravity of the same, and the quantity of the chlorides and urea in the same.

After giving the histories of the cases, he comes to the following conclusions:

1. The temperature of the body is positively heightened by the use of these preparations.

2. This increase results in some cases very soon; in one case it occurred after five hours; in others slower, and in one case after a long interval and after a large dose.

3. The temperature, the morbidly lowered as well as normal one, is increased; and if it ceases to rise after reaching a certain height, having taken a certain quantity of the iron, the temperature will rise more by increase of the dose.

4. Several days after using it the pulse rises also, although not in all cases.

5. Very soon, and consequent upon the increase of the temperature, the daily amount of urea in the urine increases.

6. The use of iron increases the weight of the body.

7. Every preparation of iron produces the same effect, and a change in the different preparations in the same patient does not alter the result.

8. The diuretic effect of citrate of iron was very distinct in two cases, but was wanting in three under the same conditions.

9. In all cases where iron was used no constipation of the bowels took place, except a slight one after iodide and lactate of iron. It was borne well, and in large doses, by the digestive apparatus (nine grains pyrophosphate of iron, and fifteen grains ferrum hydragenio reductum).

10. Dropsical transudations in the subcutaneous cellular tissue were resorbed by the use of iron, even in patients with insufficiency of the mitral valve, and reappeared after stopping with the remedy.

11. The increase of the heart's impulse and the dyspnœa in patients with organic cardiac diseases disappeared even in cases in which digitalis had done nothing.

12. After the normal temperature of the body had been raised by the use of iron, it lasted a considerable time after stopping with its use before returning to its normal condition; whilst the morbid lowered temperature rose quickly by the use of iron, it fell just as quickly by stopping with its use—at least, with the other pathological symptoms continued, and where consequently the cause of the low temperature was not cured.

Referring to these facts, the Doctor lays down the following maxims: Taking into consideration that the temperature of the body and the quantity of urea in the urine is increased by the use of iron, that the œdematus condition disappears and the weight of the body is augmented, we are fully justified in ascribing to the iron a nutritive power. The increase of temperature indicates a stronger tissue-change, for this is constant, and accompanied by other symptoms indicating a heightened nutrition. How this is brought about it is difficult to say. Increase of the blood quantum or of the blood corpuscules can not be the cause; both increase very slowly, whilst the change of tissue augments very quickly. Neither can the increase of the pulse explain the elevated temperature, as the first succeeds the latter. The respiration is not altered by the iron, hence can not have an influence upon the temperature.

According to Dr. Pakrowsky, we have, therefore, to look for the effect of iron in the finest arterial and capillary system, one of the most important places of nutrition, and the growth of the tissue and organs, and so much more, as the disappearance of dropsical transudations in the subcutaneous cellular tissue after the use of iron, points to that system. The most probable is the supposition that the iron acts upon the contractile elements of the finest arterial branches, which must have, without doubt, a high and important influence upon the capillary circulation, and, namely, upon the degree of the tonics, *i.e.*, the degree of tension of the walls of these ramifications. The iron must consequently alter the conditions of the diffusion of the elements composing the tissue and organs. Only in this way does it seem possible to explain the quick effect of iron upon nutrition and the resorption and the œdematous transudations.—*Virchow's Archiv.* xxii. 1862; *Cincinnati Lancet and Observer.*

---

Ovariotomy in Paris.—Prof. Nelaton has just performed the operation of ovariotomy, on a woman of twenty-six, who had been ill only one year, but with whom the accumulation of water was so rapid that she was tapped in May last. The cyst filled again in a short time, and M. Nelaton removed the whole growth on the 17th June. After the incision of the abdominal walls, the cyst was seized with blunt forceps and tapped. When quite empted, it was found to adhere to a portion of omentum, which latter was cut, a small portion of it remaining attached to the cyst. The latter was connected with the uterus by a very narrow pedicle, which was seized with a peculiar instrument and divided. Three small omental arteries were tied, and the wound closed by six metallic sutures. Some blood had trickled into the abdomen, and as M. Nelation considered that it might decompose, and be of great prejudice, he removed it from the cul-de-sac between the uterus and rectum by means of a sponge twice the size of a fist. The cyst was multilocular, and contained about eight quarts of viscous stringy liquid. The patient was operated on in a county house near Paris, and was in a very favorable state a week after the operation. We must not omit to state that, on the 2d of June last, Mr. Kæberle, deputy professor at the Faculty of Medicine at Strasburg, performed the operation of ovariotomy on a woman of 26 years. The diameter of the cyst was from nine to ten inches; and we are glad to say that on the 20th of June, the patient was doing extremely well, and likely to make a good recovery.—*London Lancet.*

---

PERCHLORIDE OF IRON AND INGROWING TOE-NAIL.—Dr. J. F. Miner corroborated before the Buffalo Medical Association the efficacy of this treatment. A few grains introduced by the side of the nail, between the free edge and the ulcer, was the most pleasant and efficient treatment he had ever seen adopted. He had used it in several mild cases with the greatest satisfaction; thought possibly it was better adapted to the early stages of the complaint, or to the milder degrees of irritation, inflammation or ulceration. One application was generally sufficient, when, in a few days, the condensed tissue might be removed, or, if allowed to remain, was productive of no injury; it would eventually separate, leaving a healthy surface.—*Buffalo Medical and Surgical Journal and Reporter.*

---

OZONE CORRECTIVE OF MIASMATA.—Dr. Wood gives a very important fact relative to miasmata: "These effluvia are neutralized, decomposed, or in some other way rendered innoxous by the air of large cities. Though malarious diseases may rage around a city, and even invade the outskirts, yet thay are unable to penetrate the interior; and individuals who never leave the thickly built parts, almost always escape." This he attributes to the evolution of the ethereal oils, in the combustion of wood and coal, by which a large proportion of ozone is produced.—*London Lancet.*

---

CURE OF VERICOSE VEINS.—Dr. J. F. Miner relates three cases in which he effected a complete cure, by injecting into the internal saphena a solution of the persulphate of iron, two drops, diluted with eight drops of water, thus obliterating the vein. Phlebitis has never followed this operation, the pain is less than usually attends the ligature, and the recovery is more rapid.—*Buffalo Med. and Surg. Journal and Reporter.*

